# Measurement of fasting salivary insulin and its relationship with serum insulin in children

**DOI:** 10.1530/EC-12-0024

**Published:** 2012-08-30

**Authors:** B Fabre, G Maccallini, A Oneto, D Gonzalez, V Hirschler, C Aranda, G Berg

**Affiliations:** 1 Clinical Biochemistry Department, INFIBIOC, Faculty of Pharmacy and Biochemistry University of Buenos Aires Buenos Aires Argentina; 2 Hospital Carlos G. Durand Laboratory Ciudad Autónoma de Buenos Aires Junín 956, Buenos Aires, CP 1113 Argentina; 3 TCba Salguero Laboratory University of Buenos Aires Buenos Aires Argentina; 4 Hospital Carlos G. Durand Nutrition Unit Ciudad Autónoma de Buenos Aires Junín 956, Buenos Aires, CP 1113 Argentina

**Keywords:** insulin, salivary

## Abstract

**Background:**

Saliva is a useful sample as a source of hormones for the diagnosis of different diseases, particularly in pediatric patients and aged individuals, because saliva offers a noninvasive and stress-free alternative to serum collection. The aim of this study was to validate a salivary insulin method and to check its clinical application in pediatric patients.

**Methods:**

Saliva samples were collected from 130 boys and 147 girls aged 6–14 years. Salivary and serum insulin levels were measured with the chemiluminescent automated method Access (Beckman Coulter, Brea, CA, USA). Serum blood glucose levels were measured with the glucose oxidase method in an autoanalyzer.

**Results:**

The precision profile of the method was determined for six aliquots of different concentrations from pools of saliva, and the coefficients of variation (CV) were 2.4% for 1 μUI/ml, 4% for 0.5, 8.9% for 0.25, 19% for 0.12, 28% for 0.06, and 38% for 0.03 μUI/ml, being the functional sensibility (concentration corresponding to a 20% CV) 0.12 μUI/ml. Insulin recovery was 100.13%. Salivary insulin levels diminished 29.8% in samples stored during 7 days at 2–8 °C. Differences in insulin values were not observed when samples were stored at −20 °C during 7 days. The methods used to measure salivary and serum insulin correlated significantly (*r*=0.92, *P*<0.001). However, at levels of serum insulin >20 μUI/ml, this correlation declined (*r*=0.57, *P*=0.083).

**Conclusion:**

The proposed method for salivary insulin measurement showed convenient analytical characteristics.

## Introduction

Saliva is a useful sample as a source of hormones for the diagnosis of different diseases, particularly in pediatric patients and aged individuals; the collection of saliva offers a noninvasive and stress-free alternative compared to collection of serum, for the determination of endocrine parameters. It has also an accessible cost and it is generally user-friendly, so that highly trained personnel are not required, and sample transport and storage are simple. In addition, the analysis of saliva can provide a suitable method not only for the diagnosis but also for the monitoring of the general health of large populations (1, 2, 3, 4). In recent years, there has been a significant increase in some metabolic diseases like obesity (associated with carbohydrate intolerance), type 2 diabetes mellitus, and metabolic syndrome in the population, particularly in children, all primarily due to changes in their lifestyle (5, 6, 7). Insulin that is actively transported to the saliva from its origin tissue (8) is a basic parameter to evaluate pancreatic β-cell function and clinical situations that need of additional evidence to establish a diagnosis and an appropriate therapy. The aim of this study is to validate a salivary insulin method and to determine salivary insulin levels according to age in pediatrics patients.

## Materials and methods

### Samples

Fasting blood and saliva samples were collected at 0800 h from 130 boys and 147 girls, aged 6–14 years, from two primary schools. Age, sex, weight, and height were recorded and body mass index (BMI) was calculated as weight (kilograms)/height^2^ (meters).

#### Saliva samples

The saliva samples were obtained by spontaneous salivation in sterilized tubes and samples were immediately stored at −80 °C. Upon thawing, the samples were centrifuged at 1500 ***g*** for 10 min at room temperature to sediment the heavy proteinaceous material in the samples. The supernatant was removed and an aliquot pipetted into assay tubes for the insulin assay.

#### Blood samples

Whole blood samples were collected by venous puncture after an 8 h fast. All samples were centrifuged immediately (10 min, 1000 ***g***) and the supernatant serum was stored at −80 °C until analysis. All patients gave their informed consent and the original screening study protocol was approved by the Ethics Committee of the hospital.

### Methods

Serum blood glucose levels were measured with the glucose oxidase method in a Hitachi 902 autoanalyzer, intra-assay precision (CVi) 2%, and interassay precision (CVe) 4%. Salivary and serum insulin levels were measured with a chemiluminescent automated method (CLIA) Access (Beckman Coulter, Brea, CA, USA). CLIA test is a solid-phase two-site immunoassay: one MAB is coated on the surface of the microtiter wells and another MAB labeled with HRP is used as the tracer. This test is validated for serum samples and presented a CVi for low and high levels of 6 and 3% respectively; the CVe for low and high levels was 7.5 and 3.4% respectively. The analytical sensibility of the method reported by the manufacturer was 0.03 μIU/ml (0.21 pmol/l). This method was validated in order to apply it for salivary insulin measurement.

#### Validation of the chemiluminescent method for insulin in saliva

##### Study of linearity

With the aim of evaluating the method linearity in the salivary matrix, serial dilutions of a standard solution (1 μUI/ml) were performed between 0.06 and 0.5 μUI/ml using saliva as diluent. The matrix presented an insulin concentration lower than 0.12 μUI/ml. In addition, serial dilutions of a saliva sample with a well-known insulin concentration (0.77 μUI/ml) were measured.

##### Precision profile

In order to evaluate the precision profile of the method, both intra-assay and interassay precisions were assessed in six aliquots of a pool of sera (1, 1/2, 1/4, 1/8, 1/16/, and 1/32). The former (intra-assay) was performed in quadruplicate and the latter (interassay) throughout a 10-day study.

##### Recovery and stability tests

To evaluate the recovery, an insulin standard solution (10 μUI/ml) was added to a saliva sample with known insulin concentration. The concentration of endogenous insulin in saliva was subtracted from the measured value and the result divided by the amount of added insulin. Each value was obtained in triplicate. The stability of insulin in saliva samples stored in refrigerator at 2–8 °C and frozen at −20 °C for 7 days was evaluated.

##### Correlation between serum and salivary insulin

In all cases, insulin was measured in serum and saliva with the purpose of obtaining the correlation between samples.

### Statistical analysis

Given the nonparametric distribution of the data, results are expressed as median and range values. The differences between groups were analyzed using paired *t*-test, and the correlation analysis was performed between serum and salivary insulin levels using Spearman correlations. *P* values of <0.05 were considered significant.

## Results

In the validation of this method, we performed linearity test in saliva and observed that the assay was lineal up to 1 μUI/ml of insulin. The slope for the assay of the standard solution was 0.9702 (*y*=0.9702*x*+0.0132) and for the assay of saliva samples was 1.01915 (*y*=1.01915*x*−0.0182).

The precision profile was determined for six aliquots of different concentrations from pools of saliva, and the interassay variation coefficients (CVe) were 2.4% for 1 μUI/ml, 4% for 0.5, 8.9% for 0.25, 19% for 0.12, 28% for 0.06, and 38% for 0.03, being the functional sensibility (concentration corresponding to a 20% CVe) 0.12 μUI/ml.

The mean analytical insulin recovery for 10 μUI/ml added to a saliva sample was 100.13%. The levels of insulin in saliva diminished 29.8% for samples kept during 7 days at 2–8 °C. Differences in insulin values were not observed when they were maintained at −20 °C during 7 days.

**Figure 1:**
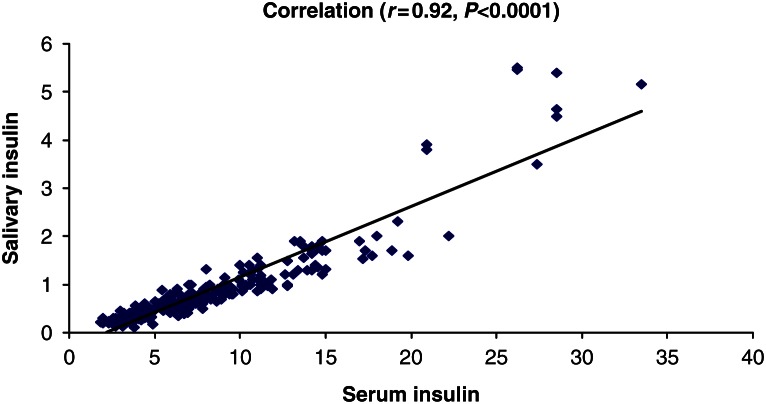
Correlation salivary and serum insulin.

When analyzing the method correlation for salivary and serum insulin, we obtained a significant correlation (*r*=0.92, *P*<0.001; Fig. 1). However, for serum insulin values higher than 20 μUI/ml, the correlation was lower (*r*=0.57, *P*=0.083).

In Table 1, the obtained salivary and serum insulin levels and BMI according to age can be observed. Among 277 studied individuals, 18 presented serum insulin levels over the normal range (15 μIU/ml).

## Conclusions

Saliva constitutes a useful tool for the evaluation of the endocrine function. The fact that saliva contains hormones was demonstrated over 40 years ago and different studies have determined that the level of diverse hormones in saliva is correlated with the level of free hormone in blood (9). To be able to use saliva as a sample for the hormonal analysis, there must be a constant and trustworthy correlation between the salivary and serum levels of the evaluated hormone. The proposed method for the insulin measurement in saliva showed convenient analytical characteristics.

The salivary measurement of hormone levels has certain advantages regarding the most conventional analyses in serum (10, 11). However, it is important to consider limitations to salivary analysis for the evaluation of hormones.

The stability of hormones in saliva is also important for their evaluation. Hormones in saliva can be degraded by enzymes, which are native of the saliva, derived from oral microorganisms or derived from the leukocytes that enter the oral cavity of the gingival furrow. These factors must be considered when saliva is being used as an alternative to serum. In this study, however, we observed that insulin stability is maintained in frozen samples.

Applying CLIA, we observed positive correlations up to 20 μUI/ml between salivary and serum insulin levels. Previous studies, applying RIA, showed positive correlations between the insulin levels in saliva and serum in healthy men (*r*=0.75) and women (*r*=0.72) (12). This was also observed in healthy individuals (*r*=0.52), in diabetic noninsulin dependable patients (*r*=0.50), and in nondiabetic obese patients (*r*=0.69), after an oral test of tolerance to the glucose (OGTT) (13). The salivary insulin levels would reach their maximum values ∼30 min after the serum levels (14). Messenger *et al*. (15) obtained lower salivary insulin levels than plasma levels (1:2) in 12 adult subjects using a RIA assay. In our study, however, applying a more sensitive method, we found that salivary insulin levels represent 10% of plasma levels. Because of the advantage of collecting salivary insulin, our method may have clinical utility for fasting insulin levels. Nevertheless, given the lower correlation obtained at levels higher than 20 μUI/ml, the use of this assay would be unuseful in OGTT or postprandial settings.

Although fasting serum insulin itself remains controversial as an indicator of insulin resistance, indices based on both fasting glucose and insulin (e.g. HOMA and QUICKI) may have more validity; more studies are needed before the same can be said of similar indices derived from saliva. This is a preliminary study that shows that salivary insulin measurements are feasible by automatized method, but more study is needed before this can be routinely used in clinical practice.

## Figures and Tables

**Table 1:** Salivary insulin, serum insulin, and body mass index (BMI) for age.

**Age** (years)	**Serum insulin** (**μ**UI/ml)	**Salivary insulin** (**μ**UI/ml)	**BMI** (kg/cm^2^)
6 (*n*=30)	4.2 (1.8–11.2)	0.4 (0.2–0.9)	16.3 (14.5–20.5)
7 (*n*=30)	5.9 (2.0–15.0)	0.5 (0.2–1.8)	16.3 (13.4–23.2)
8 (*n*=33)	4.3 (2.8–27.4)	0.4 (0.2–3.5)	16.8 (14.3–27.9)
9 (*n*=32)	7.0 (2.0–28.5)	0.8 (0.2–4.6)	18.1 (10.5–25.0)
10 (*n*=32)	4.3 (2.0–33.5)	0.38 (0.12–5.1)	18.0 (14.2–24.2)
11 (*n*=30)	8.2 (2.6–28.5)	0.75 (0.2–5.5)	19.0 (14.7–28.4)
12 (*n*=34)	10.0 (2.7–20.9)	0.9 (0.12–3.9)	19.9 (14.2–32.9)
13 (*n*=29)	7.2 (3.9–26.2)	0.8 (0.4–5.5)	18.8 (17.5–27.2)
14 (*n*=27)	9.9 (3.3–22.0)	1.0 (0.2–6.3)	20.0 (18.0–28.1)
